# Quantitative assessment of OCT and OCTA parameters in diabetic retinopathy with and without macular edema: single-center cross-sectional analysis

**DOI:** 10.3389/fendo.2023.1275200

**Published:** 2024-03-08

**Authors:** Yanyan Cui, Dongfan Feng, Changlong Wu, Ping Wang, Ruoxi Cui, Xiaokun Wang, Weiwei Chang, Weiwei Shang, Bojun Zhao, Jing Liu, Xuejiao Qin

**Affiliations:** ^1^ Department of Ophthalmology, The Second Hospital of Shandong University, Jinan, China; ^2^ Liaocheng People’s Hospital, Liaocheng, China; ^3^ Jinan 2nd People’s Hospital, Jinan, China; ^4^ Ophthalmological Center, Affiliated Hospital of Weifang Medical University, Weifang, China; ^5^ Nanchang University Queen Mary School, Nanchang, China; ^6^ Civil Aviation Medical Center of CAAC Northeast Regional Administration, Shenyang, China; ^7^ Department of Ophthalmology, Affiliated Hospital of Jining Medical University, Jining, China; ^8^ Department of Ophthalmology, Shandong Provincial Hospital Affiliated to Shandong First Medical University, Jinan, China; ^9^ Department of Biostatistics, School of Public Health, Cheeloo College of Medicine, Shandong University, Jinan, China

**Keywords:** choroidal vascularity index, diabetic macular edema, fovea avascular zone, OCT, OCTA, OCT

## Abstract

**Aim:**

The retinal and choroidal parameters were analyzed to understand the impairment of microcirculation of both retina and choroid in patients with diabetic retinopathy (DR).

**Methods:**

Fifty-five treatment-naive non-proliferative diabetic retinopathy (NPDR) patients (75 eyes) with type 2 diabetes mellitus (T2DM), including 28 patients (36 eyes) with diabetic macular edema (DME) and 27 patients (39 eyes) without DME, and 25 healthy subjects (47 eyes) were enrolled in this study. The following parameters of DR patients with and without DME were evaluated: the foveal avascular zone area (FAZ-a), FAZ perimeter (FAZ-p), FAZ circularity index (FAZ-CI), total subfoveal choroidal area (TCA), luminal area (LA), stromal area (SA), choroidal vascularity index (CVI), choriocapillaris flow area percentage, superficial capillary plexus (SCP), and deep capillary plexus (DCP).

**Results:**

SCP, DCP, and the percentage of choriocapillaris flow area were significantly different between DR patients with and without DME. The DR patients presented lower LA, CVI, and FAZ-CI compared to those of healthy controls (all p < 0.05). The percentage of choriocapillaris flow area in DR patients with and without DME was significantly lower than that in healthy controls (p < 0.05). SCP and DCP were significantly correlated with FAZ-a and FAZ-p but presented insignificant associations with FAZ-CI.

**Conclusions:**

Optical coherence tomography (OCT) and OCT angiography (OCTA) parameters, such as LA, CVI, FAZ-CI, and the percentage of choriocapillaris flow area, were reduced compared to those in controls, indicating that the microcirculations of the retina and choroid in the macular area were impaired in DR patients with DME and without DME.

## Introduction

**Figure 1:**
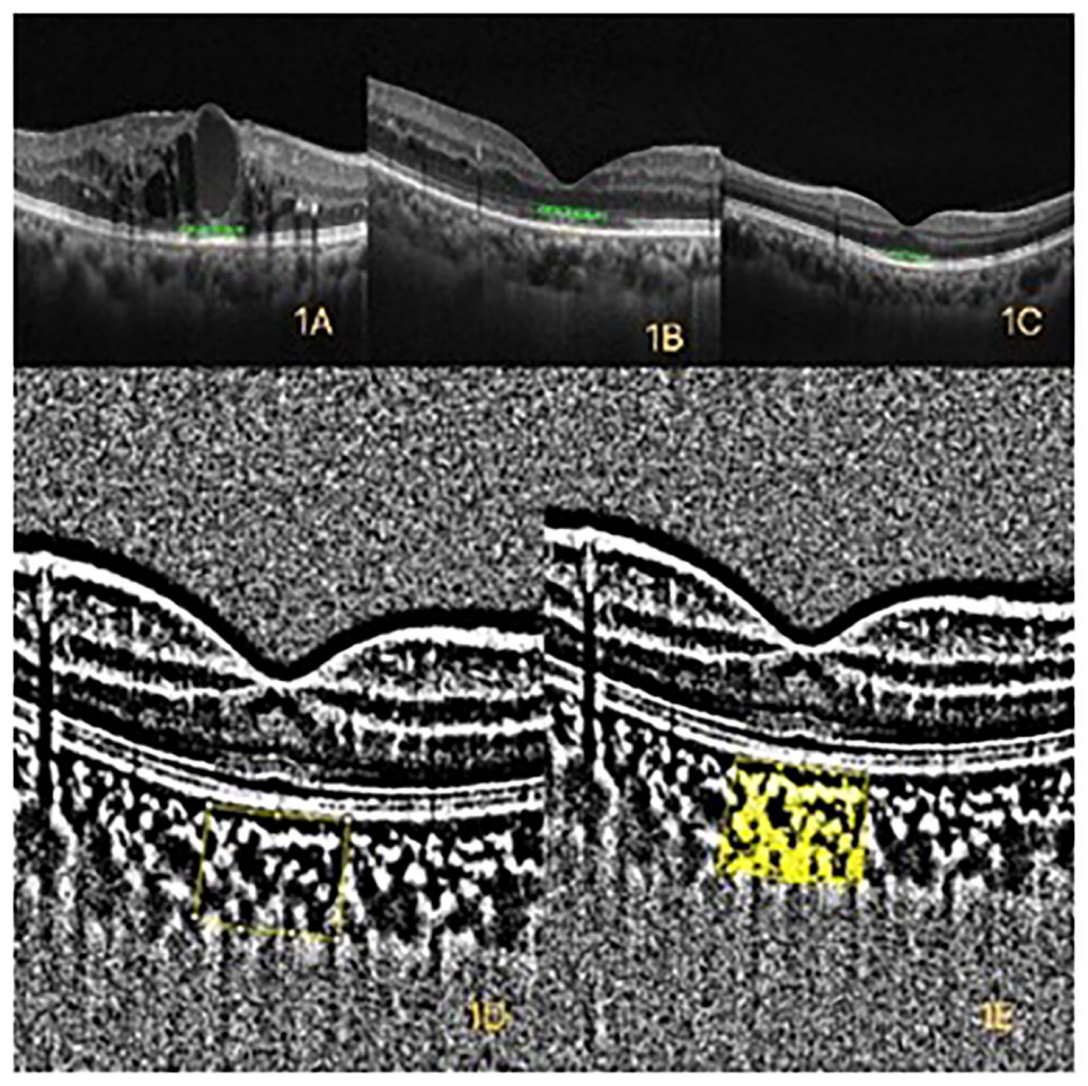
**(A)** Optical coherence tomography (OCT) of diabetic macular edema (DME). **(B)** OCT of diabetic retinopathy (DR) without DME. **(C)** OCT of healthy control. **(D)** The yellow box represents the binarized total choroidal area (TCA) within a width of 1mm in the macular. **(E)** The yellow area represents the binarized luminal area (LA) within a width of 1mm in the macular.

Patients with diabetes mellitus (DM) account for approximately 11% of the population worldwide, and DM affects 138 million adults in China (1). Diabetic retinopathy (DR) is a common complication of DM that disrupts the retinal microvasculature and a leading cause of vision loss globally (**Figure 1B**) (2). Diabetic macular edema (DME) is a common cause of vision loss in patients with DR. DME is identified by the thickening of the retina as a result of excessive fluid accumulation (**Figure 1A**) (3). The main pathophysiological event in DME is the disruption of the blood–retina barrier (BRB) caused by vascular endothelial growth factor (VEGF) and other pro-inflammatory cytokines, which in turn leads to retinal blood vessel leakage (4). The leakage may be intracellular, extracellular, or mixed. Intravitreal injection of VEGF inhibitors has recently become a major therapeutic measure for the treatment of DME (5). The choroid is an important vascular structure supplying the outer retinal layers, retinal pigment epithelium (RPE), and photoreceptors (6).

The convenience in assessing the choroid has improved with the rapid development of optical coherence tomography (OCT) and enhanced depth imaging (EDI) techniques. The choroid plays an important role in the pathogenesis of DR and DME. Histological studies have shown that DR processes affect the stroma and vasculature of the choroid (7). Vascular structures of the choroid can be classified into three layers from internal to external with the increase of luminal diameter. The innermost, middle, and outermost layers are the choriocapillaris (CC), Sattler’s layer with medium vessels, and Haller’s layer with large vessels, respectively (8). The choroidal vascularity index (CVI) has been applied to assess the vascular status of the choroid (9). The total subfoveal choroidal area (TCA; **Figure 1D**) was segmented into the luminal area (LA) and the stromal area (SA) (**Figure 1E**). The CVI was calculated as the proportion of LA to TCA (9). New techniques, such as EDI, have emerged with the rapid development of OCT techniques. Hence, the choroid can now be examined in a noninvasive manner. The CVI is a novel OCT parameter for measuring the vasculature status of the choroid (10). The CVI specifically analyzes the vascular component of the choroid, including all choroidal vessel layers, such as the CC, Sattler’s layer, and Haller’s layer. The CVI was calculated through image binarization of EDI-SD-OCT images. Agrawal et al. (9) established a normative database for the CVI among 345 healthy eyes and demonstrated that the subfoveal CVI ranges from 60.07% to 71.27% with a mean value of 65.61%  ±  2.33%. The CVI plays an important role in the early diagnosis of various retinal and choroidal diseases as well as progress monitoring (11).

The foveal avascular zone (FAZ) is typically visualized using fluorescein angiography (FA) or OCT angiography (OCTA) and a capillary-free area in the central macula with high photoreceptor density and metabolic activity. Retinal circulation is absent in the FAZ. The CC circulation is the only source of blood for the FAZ (6). The FAZ is nourished via diffusion from the underlying choroidal circulation (12), and its regular boundaries were demonstrated in the healthy person. FAZ margins can be enlarged in pathologic conditions associated with retinal capillary dropout, such as DR, and an irregular large FAZ was correlated with poor visual acuity (VA) in patients with resolved DME (13). The FAZ circularity index (FAZ-CI) was defined as the ratio of the FAZ to a perfect circle with the same perimeter as the FAZ. Samara et al. (14) revealed that both the mean FAZ-a and FAZ-CI were larger in patients’ eyes with DME than those of normal eyes; notably, the size and contour of the FAZ may vary widely among normal populations. The FAZ-CI (15) is a novel indicator and biomarker representing the disruption of the parafoveal capillary network. The shape of the FAZ is close to a regular circle when the FAZ-CI approaches 1. The FAZ-CI can be expressed as follows:


$$FAZ-CI=\frac{4\pi \times area}{perimeter^{2}}$$

Macular ischemia is usually evaluated through FA; however, the extent of macular ischemia is difficult to quantify via FA and the ischemia around the fovea may be unclear because of capillary leakage from the adjacent area. OCTA has become an essential instrument in the diagnosis and follow-up of DR in patients with and without macular edema because it allows the visualization of retinal and choroidal microvasculatures, analyses of their qualitative and quantitative changes, quantification of ischemic areas, and detection of preclinical changes.

**Figure 2:**
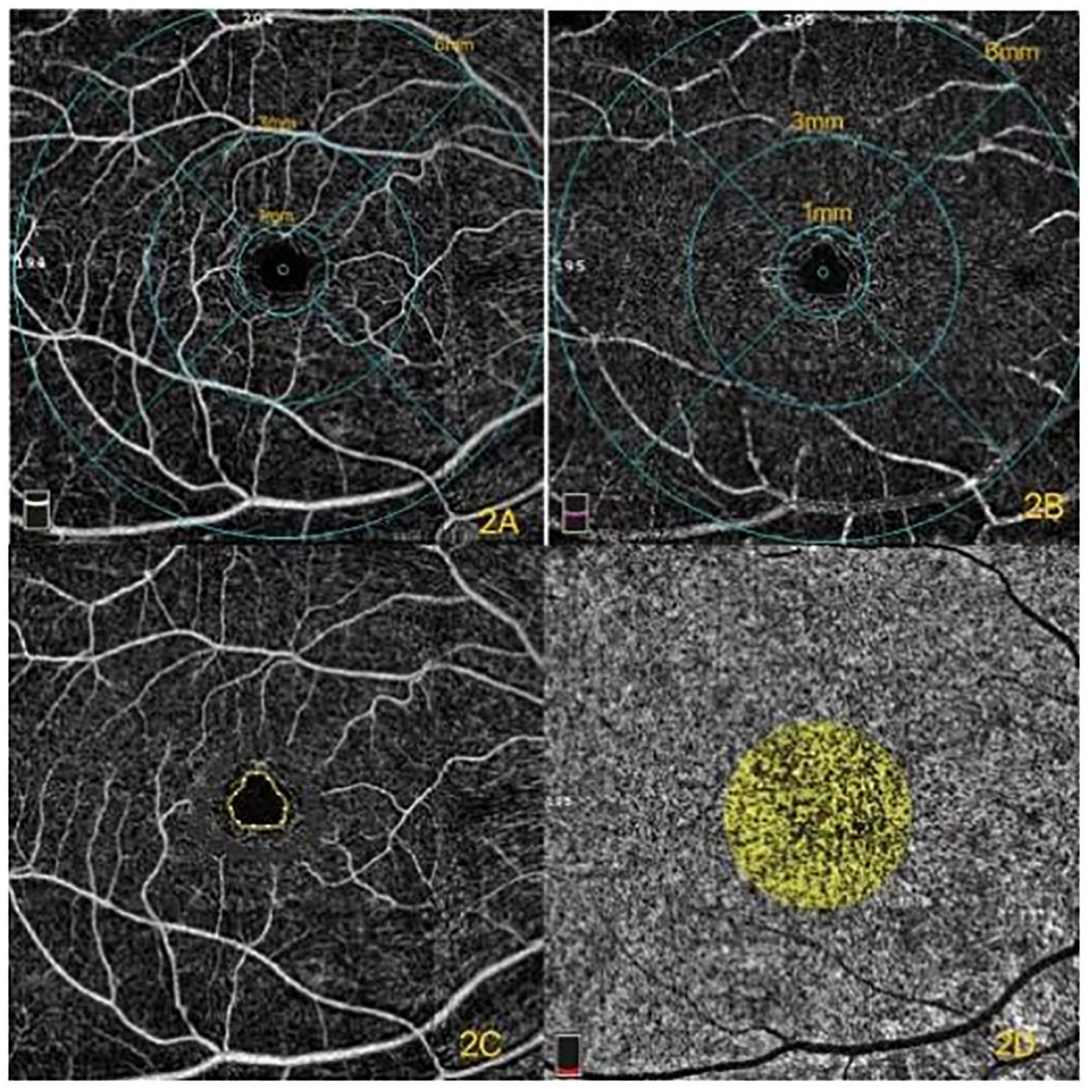
**(A)** Superficial capillary plexus (SCP) of different diameters in the macular region. **(B)** Deep capillary plexus (DCP) of different diameters in the macular region. **(C)** Foveal avascular zone (FAZ). **(D)** The yellow in the circle represents the flow of choroidal capillaries. The percentage of choriocapillaris flow area was the ratio of the yellow area to the totally selected area.

The vessel density of the macular region was measured in the annular region with an inner diameter of 1 mm (16, 17). The superficial capillary plexus (SCP) (**Figure 2A**) is formed by large and small capillaries that end at the FAZ (**Figure 2C**) as a terminate capillary ring with a centripetally branching pattern. The deep capillary plexus (DCP) (**Figure 2B**) ends at the macula with lobular patterns without direction (18).

The CC is the layer of capillaries that supplies the RPE and photoreceptors (19). Its dense network is unique compared to other capillary beds due to its large caliber and permeability. Additional details of the CC can be detected with the rapid development of OCTA. The pathologic processes of CC were able to be monitored under the examination of the OCTA images. Recent studies have demonstrated that the percentage of CC flow area (**Figure 2D**) is significantly affected by age (20). Forte et al. (21) demonstrated that CC defects were found in type 2 DM (T2DM) patients compared with those of healthy controls. This study aimed to investigate the percentage of CC flow area in DR either with or without macular edema, and the healthy subjects were the controls.

The relationship between DR and diabetic choroidopathy is still unclear. Further observing the relationship between retinal and choroidal vasculature will help us to understand the pathological progression of the disease and monitor treatment responses. Our study mainly focused on the macula area. A previous study demonstrated that the occurrence of DME is uncorrelated with the severity of DR and that DME may develop at any stage of the DR (3). The images of the eyes were captured in our study to evaluate different OCT and OCTA metrics of DR eyes with and without DME. The biomarkers, including FAZ parameters (FAZ-a, FAZ-p, and FAZ-CI), LA, SA, CVI, and the percentage of CC flow area, as well as the correlation between FAZ parameters and fovea vessel density, were comprehensively analyzed by using OCT and OCTA in this study.

## Subjects and methods

### Participants

This study followed the Ethical Principles for Medical Research Involving Human Subjects of the Declaration of Helsinki and was approved by the Ethics Committee of Shandong Provincial Hospital. Informed consent was obtained from all of the participants prior to the investigation. Fifty-five patients (75 eyes) with T2DM, including 28 patients (36 eyes) with DME and 27 patients (39 eyes) without DME, were registered in the outpatient clinic of Shandong Provincial Hospital from November 2021 to May 2022. Fourteen sex- and refractive and age-matched healthy subjects (47 eyes) composed the normal control group.

### Inclusion and exclusion criteria

Inclusion criteria were as follows: 1) participants were 40–80 years old with T2DM and 2) refractive errors between −3.00 D and +3.00 D. T2DM was defined according to the guidelines of DM from the American Diabetes Association (ADA), while DR was defined and classified by ophthalmologists according to the 2017 “Diabetic Retinopathy: A Position Statement of Diabetic Retinopathy” by the American Diabetes Association (22). Diagnostic criteria for DME included the following: 1) DR with focal or diffuse leakage in the macular area, as documented by FA, and 2) macular edema identified as center involved macular edema (central subfield thickness of 300 µm) with retinal thickening, intraretinal cyst, intraretinal hyperreflective foci, or subretinal fluid as documented by OCT The OCT of healthy control was showed by **Figuer 1C**.

Exclusion criteria were as follows: history of vitreoretinal surgery; history of macular photocoagulation; and the presence of significant epiretinal membrane, vitreomacular traction, or concurrent other ocular diseases, such as uveitis, glaucoma, optic neuropathy, eyes with VA less than 20/200 and refractive error > + 3 and < − 3, and history of intravitreal injection of anti-VEGF within 3 months. Patients with poor OCTA image quality were also excluded, such as Q-score below 5, opacity of refractive media, and the presence of significant residual motion artifacts.

### Examinations

All patients underwent a systematic history recording, detailed ophthalmic evaluation in the form of best-corrected VA (BCVA), slit-lamp examination, intraocular pressure measurement, funduscopy examination, fundus photography, and FA. Patients were diagnosed clinically significant DME according to early treatment diabetic retinopathy study (ETDRS) criteria.

OCT RTVueXR 100 Avanti instrument (Version 2017.1.0.151, Optovue, Inc., Fremont, CA, USA) verified the retinal thickness from the macular cube scan (512 × 128 pixels) to generate macular thickness map (6 × 6 mm^2^) centered at the fovea. The CVI was captured using the cross line model. Poor images including motion artifacts, poor centration, missing data, or segmentation error were ruled out, and rescanning was required.

### Imaging protocol

OCTA images for DR patients with and without DME and healthy controls were obtained in the macular region (6 mm × 6 mm) by using an RTVue XR 100 Avanti instrument (Version 2017.1.0.151, Optovue, Inc., Fremont, CA, USA). Eyes with central subfield retinal thickness (CST) greater than 300 µm or central retinal cystoid changes were considered DME. Horizontal OCT scans were binarized using Niblack threshold. The 1.0-mm-wide subfoveal choroidal area was selected as the TCA. The TCA was calculated as the polygon with the RPE as the upper boundary and sclera-choroidal junctions as the lower boundary. Dark pixels corresponding to choroidal vascular spaces were highlighted and considered the LA. The CVI was the ratio between the LA and the TCA and expressed as a percentage. The FAZ was defined as the area encompassing the central fovea without vessels under the examination of OCTA. Automated segmentation algorithms in Angio Vue software were employed to help generate segmentation lines between the superficial and deep retinal capillary plexuses.

### Image binarization details

The raster scan passing through the fovea was selected for binarization and segmented with minor modifications. Image binarization was performed using the public domain software ImageJ (version 1.47; http://imagej.nih.gov/ij/). Image binarization techniques can be applied to convert grayscale images into binarized images to facilitate tasks, such as image layout analysis and skew estimation. An appropriate image binarization technique considers that uneven illumination, image contrast variation, and poor image resolution are essential in applying a threshold to an image accurately. The LA and TCA were measured. The SA was calculated, and the CVI was determined as the LA-to-TCA ratio (11). Although concrete evidence that dark areas represent vascular areas and the light area represents stromal areas is lacking, the empirical findings of earlier studies have shown that dark areas are vascular components in binarized images (23). Only 1 mm of the macular area on the single line scan was selected as the representative segment of the macular region due to the segmental nature of the choroidal blood supply, as described by Hayreh (24).

### Statistical analysis

Statistical analyses were performed with SPSS software (SPSS 26.0, Inc., Chicago, IL, USA). Generalized Estimating Equations (GEE) was used to compensate for inter-eye correlation in statistical analysis. Descriptive statistics included numbers (percentage of each group) for nominal variables and means ± standard deviations (SDs) for continuous variables. The normality of the distribution was verified with the Shapiro–Wilk test on the basis of skewness, kurtosis values, and visual inspection of histograms. Bonferroni correction for multiple analyses was also included. Univariate linear mixed-effects models were applied to explore the correlation between random effects in DR patients with or without macular edema. A range of models with choroidal parameters as independent variables as well as age, DME+ or DME−, and healthy control covariates were created. The correlations of SCP and DCP with FAZ parameters (FAZ-CI, FAZ-a, and FAZ-p) were calculated through Spearman statistics. A p-value<0.05 was considered significant for these exploratory analyses.

## Results

### Basic characteristics of the enrolled subjects

Baseline characteristics of both groups of patients with DR and the controls are listed in **Table 1**.

**Table 1:** Basic characteristics of the enrolled subjects.

	Overall	Group			
		DR+DME+	DR+DME-	Controls	p-value
Number of patients	80	28	27	25	
Number of eyes	122	36	39	47	
Sex, female, n (%)	41 (51.25%)	13 (46.4%)	13 (48.1%)	12 (48.0%)	0.70
Age, mean ± SD	58.12 ± 6.81	57.38 ± 6.49	59.87 ± 8.08	57.29 ± 5.7	0,248
Spherical equivalent, mean ± SD	+0.51 ± 1.15	0.48 ± 1.04	0.35 ± 0.89	0.386 ± 1.28	0.487
IOP, mean ± SD	14.81± 2.15	15.11 ± 2.21	14.74± 2.52	14.58 ± 1.63	0.652

If the DR patients’ central macular subfield retinal thickness is ≥300 µm, then we denoted them as “DR+DME+.” If the DR patients’ central macular subfield retinal thickness is <300 µm, then we denoted them as “DR+DME−.” One-way ANOVA analysis, Bonferroni’s statistics, Kruskal–Wallis test, and chi-square test were applied.

Basic characteristics of the enrolled DR patients and healthy controls presented no statistical difference.

### OCT and OCTA parameters in the eyes of patients with DR and healthy controls

All DR patients with and without DME presented lower TAC, LA, SA, and CVI values compared to those of healthy controls and had statistical significance (all p < 0.05). The CC flow percentage and FAZ-CI of DR patients either with or without DME were all significantly lower than those of healthy controls (all p < 0.05). The CC flow percentage in the DME group was significantly lower than that of DR without DME (p < 0.05). The difference in outer retina flow density among the three groups had no significance. SCP in DR patients without DME was significantly lower than that of DR patients with DME and healthy controls. DCP in DR patients with and without DME was significantly lower than that of healthy controls (p < 0.05) (**Table 2**).

**Table 2:** OCT and OCTA parameters in the eyes of patients with DR and healthy controls.

Characteristics	Mean ± SE			Test of model effects		p-value for pairwise comparisons		
	Controls	DR+DME-	DR+DME+	*χ^2^*	p	DR+DME- vs. Controls	DR+DME+ vs. Controls	DR+DME+ vs. DR+DME-
CVI	0.6499 ± 0.0030	0.6374 ± 0.0027	0.6343 ± 0.0038	13.526	0.001	0.002	0.001	0.507
TCA (mm^2^)	34836.2 ± 1354.3	29972.8 ± 1925.2	30423.2 ± 1832.1	8.025	0.018	0.012	0.016	0.807
LA (mm^2^)	22590.5 ± 818.4	19073.5 ± 1179.8	19284.0 ± 1128.1	11.628	0.003	0.003	0.003	0.855
SA (mm^2^)	12247.1 ± 546.8	10897.9 ± 758.9	11142.6 ± 724.9	3.603	0.165	0.075	0.127	0.730
FAZ-a (mm^2^)	0.2829 ± 0.0214	0.3504 ± 0.0310	0.3283 ± 0.0330	4.941	0.085	0.029	0.169	0.510
FAZ-p (mm^2^)	2.0453 ± 0.0732	2.3924 ± 0.1128	2.3963 ± 0.1247	12.617	0.002	0.002	0.005	0.977
FAZ-CI	0.8211 ± 0.0101	0.7573 ± 0.0189	0.7195 ± 0.0246	23.508	<0.001	0.001	<0.001	0.170
DCP(%)	32.756 ± 1.579	26.549 ± 1.5677	29.459 ± 1.6741	7.788	0.02	0.005	0.152	0.002
SCP(%)	19.872 ± 1.536	13.998 ± 1.0293	20.533 ± 1,8219	15.581	<0.001	0.001	0.781	0.002
Choriocapillaris flow percentage(%)	0.631 ± 0.050	0.5963 ± 0.0085	0.5591 ± 0.0094	48.149	<0.001	0.001	<0.001	0.003
Outer retina flow density(%)	0.3534 ± 0.0240	0.3147 ± 0.0278	0.3036 ± 0.3337	1.872	0.392	0.291	0.228	0.799

SE, standard error; p< 0.05 highlighted; CVI, choroidal vascularity index; LA, luminal area; SA, stromal area; TCA, total choroidal area; FAZ-a, foveal avascular zone area; FAZ-p, foveal avascular zone area perimeter; FAZ-CI, foveal avascular zone circularity index; SCP, superficial capillary plexus; DCP, deep capillary plexus. The flow percentages of choriocapillaris and outer retina were the ratio of blood flow area/the selected area (3.142 mm^2^) in macular area, calculated by our machine). SCP and DCP were measured within 1 mm in diameter of the macular region.

### Correlation between FAZ (FAZ-a, FAZ-p, and FAZ-CI) and SCP, DCP

The correlations between FAZ (FAZ-a, FAZ-p, and FAZ-CI) and SCP, DCP in the three groups were analyzed. FAZ-a or FAZ-p presented a negative correlation with either SCP or DCP (all p < 0.01). Meanwhile, the correlation between FAZ-CI and SCP or DCP had no significance (**Table 3**).

**Table 3:** Correlation between FAZ (FAZ-a, FAZ-p, and FAZ-CI) and SCP, DCP.

Correlation of	DR+DME+		DR+DME-		Controls	
	Coefficient	p-value	Coefficient	p-value	Coefficient	p-value
Correlation of SCP with FAZ-p, FZA-p, FAZ-CI						
FAZ-a	-0.566	0.001	-0.556	0.001	-0.88	0.001
FAZ-p	-0.42	0.008	-0.557	0.001	-0.879	0.001
FAZ-CI	-0.142	0.42	0.193	0.245	-0.106	0.486
Correlation of DCP with FAZ-p, FZA-p, FAZ-CI						
FAZ-a	-0.686	0.001	-0.43	0.007	-0.778	0.001
FAZ-p	-0.533	0.001	-0.548	0.001	-0.794	0.001
FAZ-CI	0.031	0.85	0.336	0.039	-0.017	0.91

Coefficient: correlation index; CVI, choroidal vascularity index; LA, luminal area; SA, stromal area; TCA, total choroidal area; FAZ-a, foveal avascular zone area; FAZ-p, foveal avascular zone area perimeter; FAZ-CI, foveal avascular zone circularity index. SCP and DCP were measured within 1 mm of the macular region.

## Discussion

The correlations among quantitative OCT and OCTA parameters of DR patients with and without DME were investigated in this study. OCTA images can provide proper visualization of the microvascular architecture in intraretinal layers to demonstrate the pathological changes caused by diabetic retinal diseases quantitively (25).

The CVI is a novel OCT parameter for measuring the vasculature status of the choroid and a robust quantitative parameter of choroidal vascularity in posterior segment diseases (10). Markan et al. (26) revealed that the change in CVI even before the onset of DR supports the theory of choroidal primary damage in DR (26). Normal CVI ranges from 50% to 70%. Agrawal et al. (9) investigated the subfoveal CVI in a sample of 345 healthy eyes from subjects of the same ethnicity with a mean age of 61 years and demonstrated that the mean CVI of the subfoveal with a width of 1,500 µm is 65.61% ± 2.33%. Kim et al. (27) explored the macular CVI in five groups, namely, healthy subjects, without DR, mild/moderate NPDR, severe NPDR, and PDR, and all DR patients were diagnosed with type 2 diabetes. The researchers demonstrated that patients with diabetes had lower CVI values than those of controls and found a significant reduction of CVI from NPDR to PDR (27). Gupta et al. (28) reported that the CVI significantly decreased in DR eyes compared with that of healthy controls, and the change deteriorated from mild NPDR to PDR. Furthermore, the correlation between DME and change of CVI had no significance (28). The CVI of DR patients with and without DME in our study differed significantly from that of the healthy controls. The decrease in CVI reflects both the decrease in the number of blood vessels and the decrease in the diameter of choroidal blood vessels within a designated area. DR patients with and without DME exhibit lower CVI values than those of healthy controls, thereby reflecting that these patients presented lower choroidal vessel densities. No significant difference was observed between DR with and without DME groups. This finding is consistent with the conclusion of Gupta et al. (28) The subjects enrolled in this study were all NPDR with or without DME. The CVI between the two groups had no significance in the result of our study. Several studies had demonstrated that the CVI was associated with choroidal thickness (9, 29). Wang et al. (30) observed that the choroidal thickness reduced with the progression of the DR; in the meantime, the association between DME and the change of choroidal thickness had no significance. This finding can explain why the difference in the CVI between DR with and without DME group had no significance. Studies had reported that choroidal blood flow decreased in patients with diabetes, especially in PDR patients (31). Our results showed that the LA, TCA, and CVI of DR patients with and without DME differed significantly from those of healthy controls. Our finding is consistent with other studies indicating the impairment of the CVI in DR patients. The CVI indirectly and quantitatively reflects the condition of choroidal vascularity and overcome the limitation of using choroidal thickness alone. Moreover, the CVI can be used to analyze the impairment of choroidal vascularity and as a follow-up tool for treatment response. The SCP and DCP within a diameter of 1 mm in the macular region were measured in this study. Several studies had assessed the vascular dropout and changes in vascular density (VD) in DR, and majority of the studies had shown that the retinal VD significantly decreased with the progression of DR (32, 33). Dimitrova et al. demonstrated that SCP and DCP in the parafoveal region decreased and the significant enlargement of FAZ in diabetic patients with DR and even in those diabetic patients without DR compared that of healthy controls (34, 35). The SCP and DCP reduced significantly in mild NPDR compared with that of healthy controls (36). Kim et al. (37) revealed that the VD in patients with severe NPDR and PDR was significantly lower than that in patients with mild NPDR and normal controls; furthermore, patients with DME demonstrated significantly lower VD values. In our study, the comparison between DR and healthy controls revealed that DR without DME presented statistically significantly lower VD values in SCP and DCP. The result was similar to that of a previous study (38). VD was lower in DR patients than that of healthy controls; it is likely due to the following reasons. First, the hyperglycemia environment promotes the damage of the BRB and degeneration of microvascular endothelial cells (39). Second, exudates in the retina can compress peripheral capillaries around the FAZ, affect tissue metabolism, and further aggravate capillary occlusion and degeneration (3). Some studies have demonstrated that VD is negatively correlated with systemic factors, such as fasting blood glucose, postprandial blood glucose, and HbA1c, but other studies have shown that VD is not associated with HbA1c or the duration of the disease (32, 40, 41). Hence, further experimental and clinical investigations are needed to explore the contributing factors for VD in DR with and without DME patients.

The choroid is responsible for the blood supply of the outer retina, including RPE and photoreceptors, and is the only source of metabolic exchange for the avascular fovea (42). Diabetic choroidopathy includes CC dropout, luminal narrowing and obstruction, and microaneurysms (43, 44). Nesper et al. (45) used OCTA to evaluate the nonperfusion area in the CC layer of diabetic eyes and revealed that the nonflow areas were enlarged in diabetic eyes. Choi et al. (46) qualitatively demonstrated impairment of the CC flow using a swept-source OCTA prototype, although motion errors remained uncorrected. The capillary flow density (CFD) of the CC in DR patients of both with and without DME was significantly lower than that of healthy controls in our study. This finding is similar to the results of a previous study (45). Previous histopathological studies on diabetic eyes demonstrated the secretion of VEGF involved in the degeneration of the CC layer with loss of endothelial cells, obstruction, and choroidal aneurysms as well as degeneration of RPE ultimately resulting in the formation of choroidal neovascularization (47). The level of VEGF increases with the development of DR, especially in eyes of PDR with DME (48, 49). Therefore, the decreased CFD in the CC layer may be associated with the increase of VEGF. The CC flow density of DR with DME is significantly lower than that of DR without DME, while the CVI between them is nearly the same. We consider that is caused by retinal vascular dysfunction and vasculitic dilation in DME patients. A previous study demonstrated that CFD was associated with coronary artery disease, atherosclerosis, hypertension, and levels of lipid metabolism, HbA1c, and eGFR (50). The CFD not only represents the status of choroidal capillary but also reflects the patient’s systemic conditions. In summary, the general conditions should be carefully assessed in diabetic patients with a significant decrease of CFD. Further experimental and clinical investigations are needed to explore the affect factors for CFD in DR with and without DME patients.

FAZ-p, FAZ-a, and VD are effective signals for reflecting the degree of retinal ischemia that have been applied in several clinical studies for predicting disease progression (51). A previous study demonstrated that the FAZ was significantly larger in the DR group than that in healthy controls (52). Di et al. (53) demonstrated that the FAZ was significantly larger in the clinically significant macular edema (CSME) group vs. the non-CSME group in a singular plexus. Meanwhile, Freiberg et al. (54) and Takase et al. (55) examined both the SCP and DCP and showed that the FAZ in the deep plexus increased more significantly in DR patients than that in healthy eyes and the FAZ significantly enlarged even in DM patients without DR. A previous study also demonstrated that the enlargement of the FAZ-p in the superficial and deep plexus was correlated with DR severity as well as the duration of diabetes (56). Krawitz et al. (56) observed differences in the FAZ shape between DR patients and controls and demonstrated the absence of differences in DM patients without DR compared to that of healthy controls. Another study also showed that the FAZ of patients with DM but without DR and healthy controls had no difference (57). An enlarged FAZ associated with the reduction in the VD of the SCP and DCP within 1 mm of the macular region was observed in patients with NPDR (58). A previous study demonstrated that a large FAZ was correlated with poor VA in patients with resolved DME (13). Samara et al. (59) also indicated that a large FAZ-a and poor VD in the macular area were associated with poor VA in patients with DR. Ragkousis et al. (60) demonstrated that the difference in the FAZ and perimeter between NDR and controls had no difference, while they differed significantly between NDR and mild NPDR groups. Our study presented that either FAZ-a or FAZ-p was negatively correlated with both superficial and deep plexuses in the 1-mm macular region. This finding is consistent with the results of a previous study (58). Our results showed that DR patients without DME presented statistically significantly larger FAZ-a than that of healthy controls, while the size of the FAZ had no statistical difference between DR with DME and healthy control. However, our results on the FAZ between DME and healthy controls were different from those of a previous study (58). We consider no difference of the FAZ between DR with DME and healthy control may have the following reasons: first, the fluid in the intraretinal and subretinal changed the formal shape of the FAZ. In addition, DME might induce artifacts and thus affected the accuracy of the results. Furthermore, the decay of the image signal intensifies when the light source penetrates deep in the retina in the DME group. The FAZ can be used to predict not only the severity of the vessel dropping out but also the change of the VA in DR patients after treatment.

The circularity index provides information about the damage of the FAZ in pathological conditions. Compared with the FAZ-a, the FAZ-CI is a stable measurement index for assessing FAZ circularity with a value range of 0–158. The FAZ-CI in DR of both with and without DME groups exhibited a lower index than that of healthy controls in our study. In the meantime, the mean FAZ-a values were larger in the eyes of DR patients either with or without DME than those of normal eyes. The results are similar to those of Hsieh et al. (61). Meanwhile, the association between the FAZ-CI and SCP or DCP had no significance. To the best of our knowledge, the present study is the first to investigate the change of FAZ-CI values in DR patients. Our results demonstrated that FAZ-CI values in DR patients either with or without DME were lower than those of healthy people; it is likely due to the enlargement of FAZ-a and FAZ-p, especially the increase of FAZ-p, in DR patients. The difference of the FAZ-CI between DR patients with and without DME had no significance. Thus, investigating the FAZ-CI is important for clinicians to understand the impairment of the microcirculation of DR patients.

The study showed that the microcirculation in DR patients was damaged to some extent, and ophthalmologists should pay attention to the microcirculation of DR patients. This study can also help clinicians evaluate the fundus microcirculation status in patients with DR based on OCT and OCTA indicators.

This study had several limitations. First, macular edema itself may affect the signal strength of the OCTA. Second, as mentioned previously, projection artifacts may affect the imaging quality of the deep retinal layer than that of the superficial layer. Finally, the number of participants enrolled in this study was limited. The accuracy of prediction for treatment outcomes of DME can be improved in future investigations with the advent of more advanced OCTA equipment.

## Conclusions

The relationship between FAZ metrics (FAZ-a, FAZ-p, and FAZ-CI) was explored in this study. FAZ-a or FAZ-p with either superficial or deep fovea vessel density was negatively correlated. The total TCA, LA, CVI, and choroidal capillary flow significantly decreased in DR patients with and without macular edema. OCT and OCTA can help clinicians to understand the impairment of the microcirculation in DR patients and follow up the treatment efficacy.

## Data availability statement

The original contributions presented in the study are included in the article/supplementary material. Further inquiries can be directed to the corresponding author.

## Ethics statement

The studies involving humans were approved by The ethics committee of Shandong provincial hospital. The studies were conducted in accordance with the local legislation and institutional requirements. The participants provided their written informed consent to participate in this study.

## Author contributions

YC: Conceptualization, Data curation, Investigation, Project administration, Visualization, Writing – original draft, Writing – review & editing. PW: Investigation, Writing – review & editing, Supervision. DF: Writing – review & editing, Data curation, Project administration. XW: Data curation, Writing – review & editing, Methodology. CW: Writing – review & editing, Project administration. WC: Formal Analysis, Software, Writing – review & editing. WS: Investigation, Project administration, Writing – review & editing. BZ: Validation, Writing – review & editing. XQ: Funding acquisition, Resources, Visualization, Investigation, Writing – review & editing. RC: Data curation, Writing – review & editing, Methodology. JL: Formal analysis, Software, Writing – review & editing.
